# Familial confounding or measurement error? How to interpret findings from sibling and co-twin control studies

**DOI:** 10.1007/s10654-024-01132-6

**Published:** 2024-06-16

**Authors:** Kristin Gustavson, Fartein Ask Torvik, George Davey Smith, Espen Røysamb, Espen M. Eilertsen

**Affiliations:** 1 Department of Psychology, University of Oslo, Oslo, Norway; 2 Norwegian Institute of Public Health, Oslo, Norway; 3 Centre for Fertility and Health, Norwegian Institute of Public Health, Oslo, Norway; 4 MRC Integrative Epidemiology Unit, University of Bristol, Bristol, UK; 5 Promenta Research Centre, Department of Psychology, University of Oslo, Oslo, Norway

**Keywords:** Sibling control studies, Confounding, Co-twin control, Simulation studies

## Abstract

Epidemiological researchers often examine associations between risk factors and health outcomes in non-experimental designs. Observed associations may be causal or confounded by unmeasured factors. Sibling and co-twin control studies account for familial confounding by comparing exposure levels among siblings (or twins). If the exposure-outcome association is causal, the siblings should also differ regarding the outcome. However, such studies may sometimes introduce more bias than they alleviate. Measurement error in the exposure may bias results and lead to erroneous conclusions that truly causal exposure-outcome associations are confounded by familial factors. The current study used Monte Carlo simulations to examine bias due to measurement error in sibling control models when the observed exposure-outcome association is truly causal. The results showed that decreasing exposure reliability and increasing sibling-correlations in the exposure led to deflated exposure-outcome associations and inflated associations between the family mean of the exposure and the outcome. The risk of falsely concluding that causal associations were confounded was high in many situations. For example, when exposure reliability was 0.7 and the observed sibling-correlation was r = 0.4, about 30–90% of the samples (n = 2,000) provided results supporting a false conclusion of confounding, depending on how p-values were interpreted as evidence for a family effect on the outcome. The current results have practical importance for epidemiological researchers conducting or reviewing sibling and co-twin control studies and may improve our understanding of observed associations between risk factors and health outcomes. We have developed an app (SibSim) providing simulations of many situations not presented in this paper.

**Supplementary Information:**

The online version contains supplementary material available at 10.1007/s10654-024-01132-6.

## Introduction

Epidemiological researchers often aim to answer questions about causal relationships. Do childhood traumas cause depression? Does maternal medication use during pregnancy cause neurodevelopmental disorders in the child? Do mental health problems impact low back pain? However, causal inferences must often rely on unverifiable assumptions when exposure-outcome associations are estimated from non-experimental designs. For example, the personality trait conscientiousness is associated with longevity [1], but this association could be confounded by genetic factors. Childhood trauma is associated with depression in adulthood [2], but this association might be confounded by other characteristics of a person’s conditions when growing up (e.g., parental mental health). Researchers can control for some confounding factors by including them in the study. However, there may always be other factors that are not measured, or potential confounding factors may be poorly measured and thus not properly accounted for even if they are included [3].

Sibling control designs are often used to account for unmeasured confounding factors that are shared among family members [4, 5]. Twins are siblings, and a co-twin control design with monozygotic twins may be particularly effective for controlling for genetic confounding factors since monozygotic twins share all their germ line genome [4]. If the association between mental health problems and low back pain is entirely genetically confounded, a co-twin control design with monozygotic twins would typically find that the twin with more mental health symptoms does not have higher chance of getting low back pain than the twin with less mental health symptoms. Studies using dizygotic twins or other siblings can normally only account for half of the genetic variation [4]. However, twins and other siblings also share environmental factors. Siblings that are not twins have been in the same womb at different times. Sibling designs have therefore been frequently used to control for stable maternal confounding effects (such as maternal personality and maternal genetics) when examining the effect of maternal health behaviour during pregnancy on child health outcomes [6]. For example, there is an association between maternal long-term acetaminophen use in pregnancy and ADHD in the child [7]. When a mother used acetaminophen for many days in one pregnancy and not in another, the sibling exposed to long-term acetaminophen use should have higher risk of ADHD than the unexposed sibling if acetaminophen use is causally related to ADHD. If the association is confounded by for example maternal genetic risk for ADHD (e.g., the mother has increased risk of using acetaminophen during pregnancy because of her own ADHD traits), the two siblings are not expected to differ regarding risk of ADHD as they will have the same risk of inheriting the maternal genes for ADHD regardless of whether they were exposed to acetaminophen during pregnancy or not [8].

Sibling designs have been very useful for investigating the nature of exposure-outcome associations. A common practice for interpreting these designs can be summarized as follows: When an association between two phenomena is established among unrelated individuals (e.g., between conscientiousness and health), this association can be further investigated among twins or other siblings to check for unmeasured familial confounding factors. If the association is causal, it is expected to also appear when comparing siblings in the same family.

Previous research has discussed different limitations and sources of bias in sibling control designs [4, 5, 9–14]. One important source of bias is measurement error in the exposure variable, which attenuates association estimates more when comparing siblings in the same family than when examining associations in unrelated individuals [5, 9]. This is because two family members’ exposure variables (e.g., their level of conscientiousness) tend to be correlated. The higher the correlation between the two exposure variables, the more the association between the exposure and the outcome will be attenuated due to measurement error in the exposure variable [5]. This implies that measurement error may lead to a pattern of associations that will normally be taken as evidence for familial confounding in a sibling control study, even if the association is truly and entirely causal [5]. The following example may illustrate the point: If the association between maternal acetaminophen use in pregnancy and child ADHD is truly causal, but maternal acetaminophen use is measured with error, the association will appear as weaker when comparing discordant siblings than when the association is examined in a sample of unrelated individuals. Hence, the sibling-control model can be more biased than the model not comparing siblings. Sibling control studies can be very useful, but their results must be interpreted with sufficient information about the potential consequences of measurement error in mind [12, 15, 16]. As monozygotic twins share 100% of their genes, their exposure variables may be more highly correlated than dizygotic or other siblings’ exposures. Effects of measurement error may thus be particularly high in studies comparing monozygotic twins. For example, the association between mental health symptoms and low back pain may be present when comparing siblings or dizygotic twins, but absent when comparing monozygotic twins [17]. It may then not be clear what estimate is the least biased: The one from comparing monozygotic twins because of more effective control for familial confounding, or the one from comparing dizygotic twins or other siblings because monozygotic twins’ exposure variables are more highly correlated, and thus more heavily affected by measurement error.

## Researchers need information on how to interpret their findings

Even though measurement error is a known source of bias in sibling control studies, it may be difficult to know the consequences of this insight when planning such studies or interpreting findings. Simulation studies are ideal for investigating effects of different kinds of biases because obtained results can be compared to known population values [18, 19]. The current study will therefore use Monte Carlo simulations to examine attenuation of association estimates in sibling control models due to measurement error when the observed exposure-outcome association is truly causal. By studying varying levels of measurement error and varying degrees of observed correlations between siblings’ exposure variables, we will provide information to which researchers can compare their results from sibling control studies, to evaluate if their results should be interpreted as evidence for familial confounding or as due to measurement error.

### Quantification of the risk of drawing false conclusions about familial confounding

We are not aware of any studies providing researchers with quantifications of the risk of falsely concluding that a true causal effect is confounded in sibling control studies. The estimated association between the family mean of the exposure and the outcome, controlled for the individual’s exposure variable, may be interpreted as a reflection of familial confounding-that is, the effect of the family on the outcome. The p-value of this estimate may help the researcher determine the strength of the evidence for familial confounding. However, relying on an arbitrary cut-off, such as *P* < 0.05, to categorize p-values as significant or non-significant, may be highly problematic because there are several aspects of a study that need consideration when interpreting p-values [20]. We will present precise p-values to demonstrate that they need to be interpreted in the context of exposure reliability and sibling-correlations in the exposure. The proportion of simulated samples with p-values below a certain cut-off value will represent the risk of falsely concluding that confounding exists when using that cut-off as evidence for the association between the family exposure mean and the outcome. For example, in a situation where 2% of the simulated samples provide a p-value below 0.01 for this association, there will be a two percent risk of drawing a false conclusion of familial confounding if *P* < 0.01 is interpreted as strong evidence for the association in that situation. If 20% of the samples provide a p-value below 0.05, the risk of drawing a false conclusion of familial confounding will be 20% if the researcher decides that p-values below 0.05 is strong evidence of the existence of familial confounding. To offer researchers a frame of reference for interpreting p-values in their own studies, we will present findings from various simulated scenarios with different levels of exposure reliability and sibling correlations. Sample size greatly impacts p-values, and we will therefore include results from simulations conducted with varying sample sizes.

### Choice of analytical approach depends on the outcome variable

**Fig. 1:**
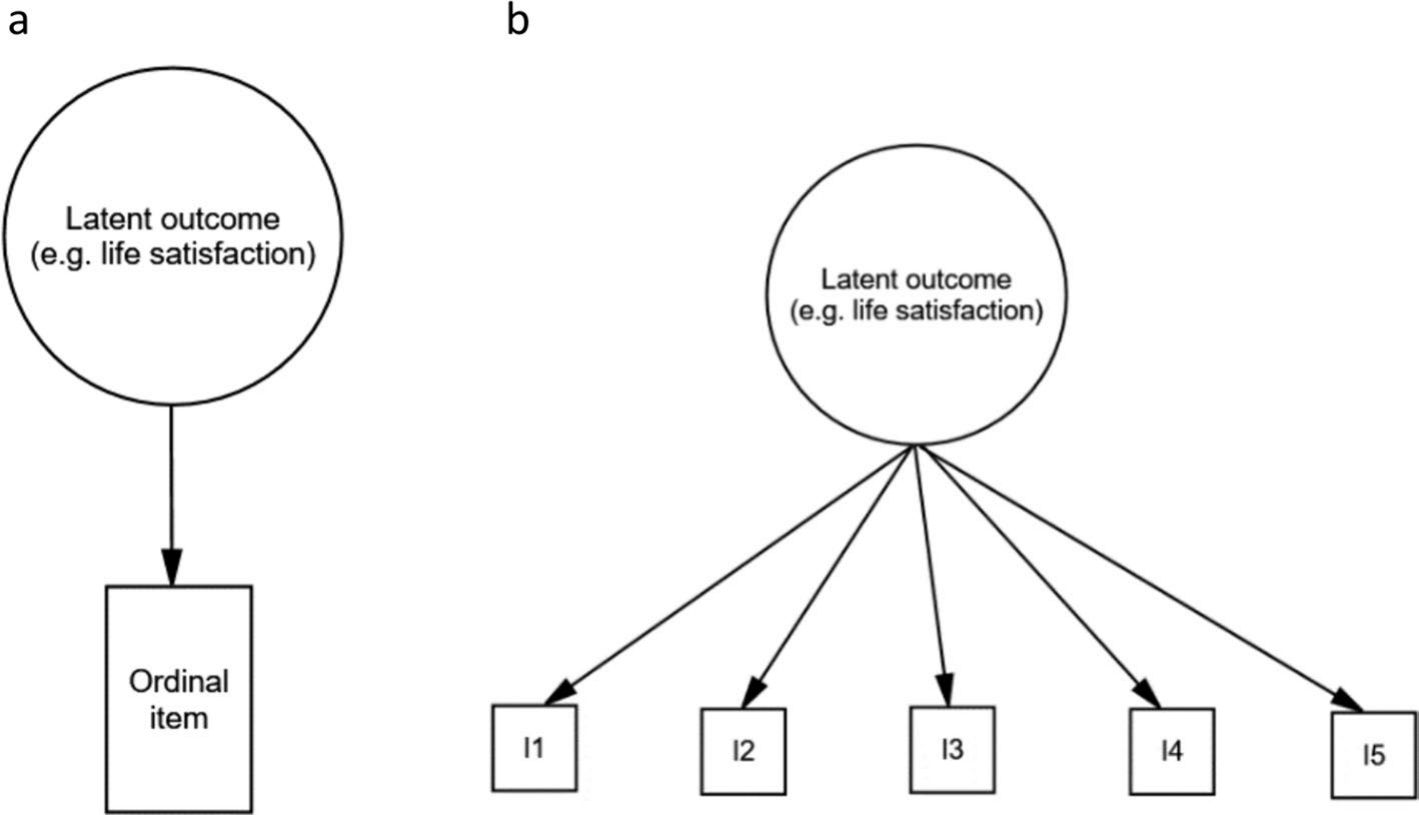
Latent factor models. Notes: The circles represent latent traits that are not directly measured. Squares represent ordinal questionnaire items. I1 to I5 = Item1 to Item5

As discussed above, the main aim of the current study is to examine effects of measurement error in the *exposure* variables. However, it is also important to address measurement of the outcome variable, as this has implications for the choice of analytic model. Frisell and colleagues [5] have previously shown the effects of measurement error in the exposure variable when using linear models with continuous outcomes as well as logistic models with binary outcomes. The deflation of association estimates in linear sibling control models with continuous outcomes can be calculated, and thus also corrected [5]. However, linear regression models may not fit well with outcome variables measured with ordinal Likert scales in questionnaires (e.g., a five-point scale ranging from “Strongly disagree” to “Strongly agree”) [21–24]. Some studies use only one Likert scale question as outcome, such as a single-item life satisfaction measure [25]. The latent trait (e.g., life satisfaction) is not measured directly, but will affect how people respond to the ordinal questionnaire item. This is illustrated in Fig. 1a. This ordinal level outcome variable requires an appropriate statistical model [22, 26]. The current study will expand our understanding of the effects of measurement error in exposure variables in sibling control studies by using ordered probit regression models with ordinal outcome variables. More details on the ordered probit model and its application in simulation studies are discussed elsewhere [22, 27].

Often, the outcome in questionnaire studies is an aggregate score of several Likert-scale items (see Fig. 1b). A mean or sum score of the ordinal items can be calculated and used as a continuous outcome variable in linear models. However, this outcome variable is not truly continuous, and the performance of linear models with such outcomes needs to be examined.

### The current study

The main aim of the current Monte Carlo simulation study is to enhance our understanding of the impact of measurement error in the exposure in sibling control studies. Specifically, we will achieve this by: 1) Exploring how truly causal exposure-outcome associations are attenuated only because of measurement error. This will be done for several different situations, including different levels of exposure reliability and sibling-correlations in the exposure, when using single-item ordinal outcome variables, as well as aggregates of several ordinal variables, and 2) Quantifying the risk of falsely concluding that familial confounding is present in different situations. We will show the importance of considering exposure reliability and sibling correlations in the exposure when interpretating p-values as evidence for familial confounding. The findings will have practical implications for researchers who conduct or review sibling control studies, consequently enhancing the utility of such study designs. Additionally, we have developed a user-friendly Shiny app called SibSim, which enables researchers to experiment with different conditions and explore numerous situations and combinations of factors that are not covered in the paper. This includes varying sample sizes, sibling correlations in the exposure, exposure reliability, effect sizes of exposure on outcome, type of outcome variable, and levels of asymmetry in ordinal outcome variables.

## Single-item ordinal outcome

### Methods

**Fig. 2:**
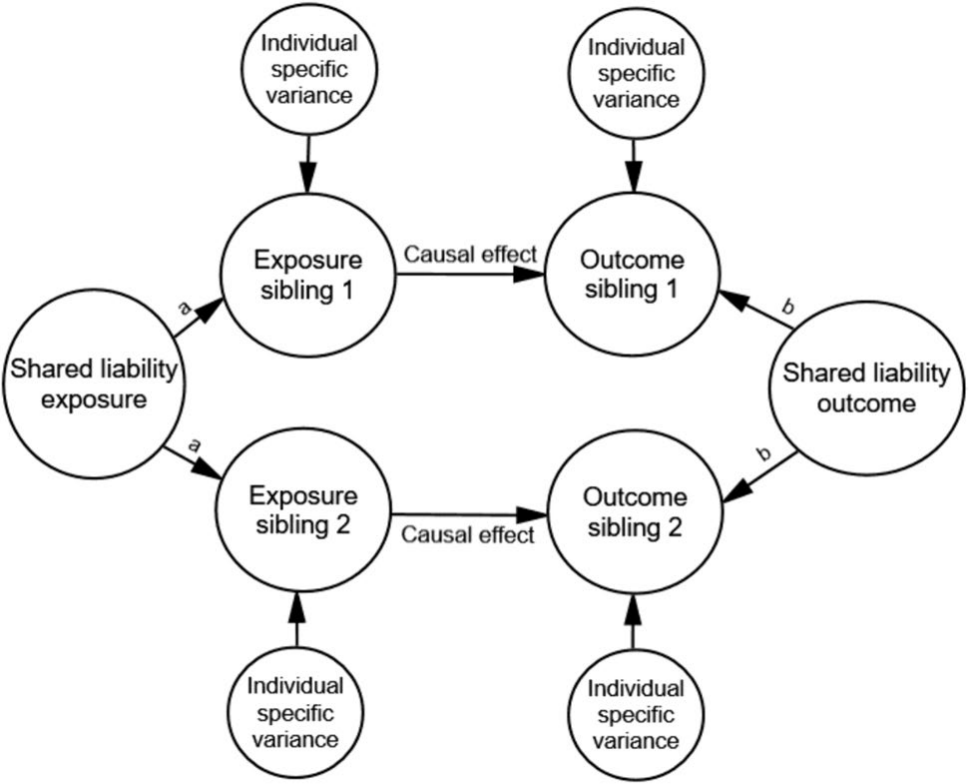
Illustration of the population the data were generated from. Notes: The circles represent population phenomena that are not directly measured. There is no shared confounding as there is no association between the latent variables “Shared liability exposure” and “Shared liability outcome”. There are no associations between individual specific variance for exposure and outcome, implying no individual-specific confounding

Data were simulated with the MASS package [28] in RStudio [29]. Populations were defined with normally distributed continuous exposure and outcome variables (mean = 0, variance = 1) for two siblings in each family. The populations differed regarding the level of correlation between the two siblings’ exposure variables and regarding the strength of the causal relationship between the exposure and the outcome. See Fig. 2 for an illustration of the population model. There was no shared confounding in the population (no association between the shared liability of exposure and outcome) because the aim of the current study was to examine attenuation of association estimates in sibling control models due to measurement error when the observed association is truly causal.

From each population, 500 random samples were drawn, each consisting of 500; 2,000; or 5,000 sibling pairs. Varying degrees of measurement error (0%, 10%, 20%, 30%, and 40% of total variance) were induced by adding random elements to the exposure variables. These random errors were normally distributed and uncorrelated between siblings and uncorrelated with other variables within siblings. The variables with measurement error will be termed “observed variables”. Reliability of the observed variables is defined as true variance divided by total variance and varied between 1 and 0.6.

As shown by Frisell and colleagues [5], the stronger the correlation between the two siblings’ exposures, the larger is the effect of measurement error. Empirical studies have shown correlations of 0.8, 0.7, and 0.6 for two monozygotic twins’ risk of having bipolar disorder, schizophrenia, and any mental or behavioural disorder, respectively [30], and correlations up to about 0.6 between siblings’ similarity in educational attainment, school grades and cognitive skills across different countries [31]. In the current study, we used a wide range of different observed exposure correlations (from 0.2 to 0.6) in the main analyses, and we also included additional analyses of data with exposure correlations of 0.7, 0.8, and 0.9, to provide relevant information for studies using monozygotic twins with highly correlated exposure variables. By focusing on the observed, rather than true, exposure correlations, the situations are more intuitively relevant for comparison with real data. True exposure correlations are the observed correlations divided by the reliability of the exposure.

Measured ordinal outcome variables were generated by categorizing the continuous outcome variables into five response categories, reflecting a model where ordinal variables represent underlying continuous phenomena [22, 27, 32, 33]. Symmetric outcome variables were modelled in this paper, while the SibSim app also allows modelling and analysing moderately and highly asymmetric outcomes. Table 1 shows the proportions of responses in each of the five categories for different levels of symmetry/asymmetry.

**Table 1:** Proportions of responses in the five categories at varying levels of asymmetry in the ordinal outcome variables

	Symmetric	Moderately asymmetric	Highly asymmetric
Proportions:	3.6%, 23.8%, 45.2%, 23.8%, and 3.6%	75.2%, 9.1%, 6.8%, 5.1%, and 3.8%	90.0%, 4.0%, 3.0%, 2.0%, and 1.0%
Available:	In the paper and the SibSim app	In the SibSim app	In the SibSim app

As discussed above, single-item outcome variables were analysed with an ordered probit regression model. The initial model was run with the polr function from the MASS package, treating the outcome as an ordered categorical variable [28]. The sibling control model was run by adding the siblings’ mean value of the exposure to the former model. The probit link provides a quite straightforward interpretation of the regression coefficient (i.e., the association between the predictor and the underlying continuous normally distributed latent outcome variable). This fits well with how the data were modelled. In probit regression, the residual variance of the underlying continuous latent outcome variable is fixed to 1 [34]. This implies that total variance increases when explained variance increases (for example by adding an extra predictor variable to the model), which may re-scale the regression coefficient. All regression coefficients from probit regression models were therefore standardized with respect to the underlying continuous response variable [27, 35]. See Supplementary Information for details on this.

### Results

**Fig. 3:**
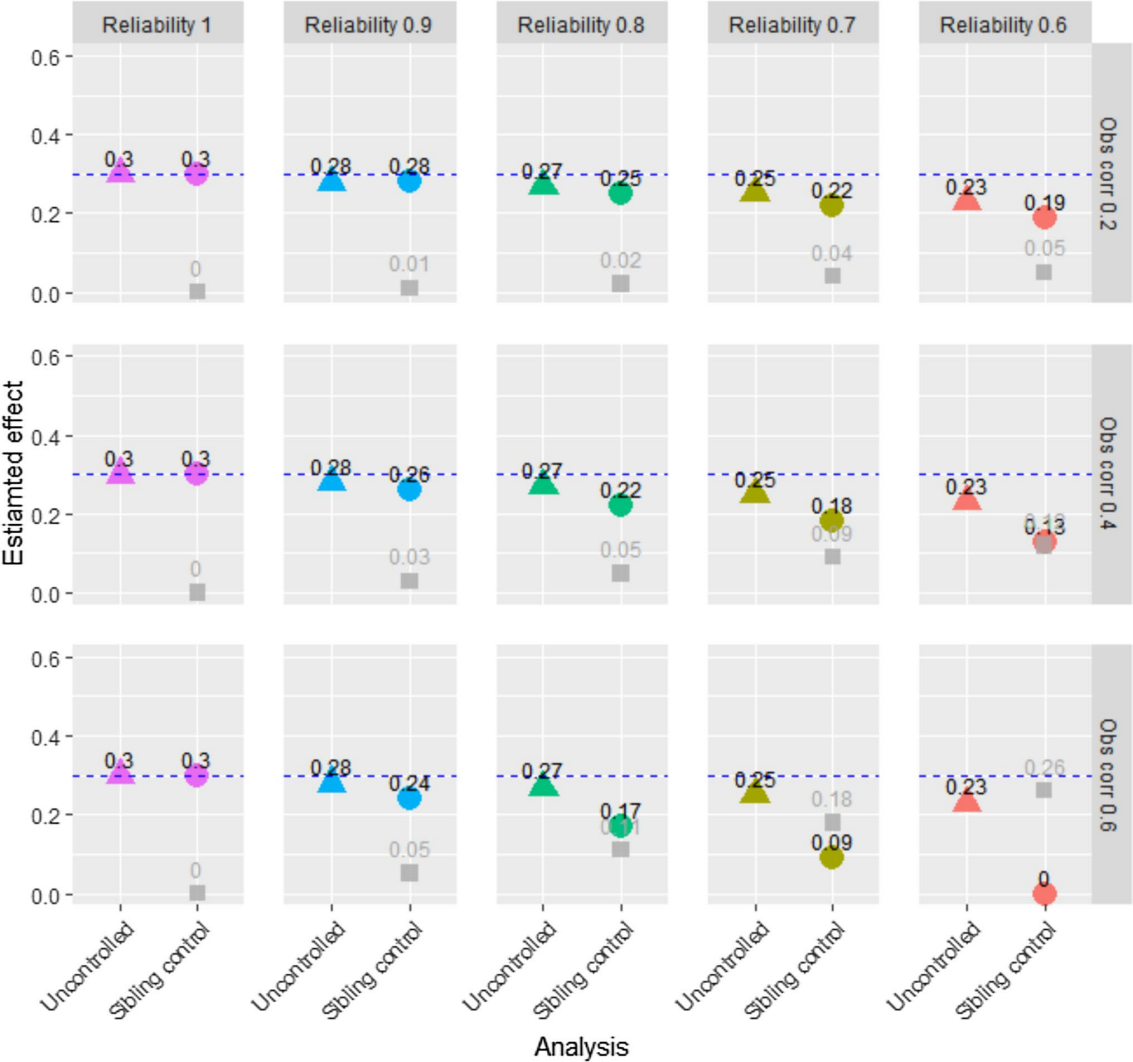
Results from uncontrolled and sibling control models—ordinal single-item outcome—n = 2,000. Notes: “Obs corr” is the observed correlation between two siblings’ exposure variables. “Reliability” is the reliability of the exposure variables. The outcome is a five-level symmetric single-item ordinal variable. Proportions in the five response categories of the outcome were: 3.6%, 23.8%, 45.2%, 23.8%, and 3.6%. The true causal effect was b=0.3, as indicated by the dotted line. Triangles represent the estimated association between the exposure and the outcome in an uncontrolled model. Circles represent the estimate of this association in the sibling control model. Gray squares represent the estimated association between the family mean of the exposure and the outcome. The true value of this latter association is zero

Figure 3 shows results from uncontrolled and sibling control models at different levels of reliability of the exposure variables and different levels of correlation between the two family members’ exposure variables. The true causal effect of the exposure on the outcome was b = 0.3, and sample size was 2,000. The outcome variable was a symmetric single-item ordinal variable with five response categories. Figure 3 shows that when the exposure variable was measured with perfect reliability, uncontrolled and sibling control models both gave unbiased results of the causal effect of the exposure on the outcome. As reliability decreased, the estimates from the sibling control model decreased more than the estimates from the uncontrolled model. When the correlation between the two family members’ exposure variables increased, the effect of measurement error on the sibling control model increased. Hence, as reliability decreased and exposure correlations increased, the results from uncontrolled and sibling control models became increasingly different, even though there was no confounding. When reliability was 0.6, and the observed exposure correlation was 0.6, the true correlation between the siblings’ exposure was 1.0. Hence, there was no true difference in the exposure between siblings, and the sibling control model estimated a zero association between exposure and outcome. The grey squares in Fig. 3 represent the estimated association between the family mean of the exposure and the outcome. This estimate may be seen as evidence for familial confounding, and its true value was zero. Figure 3 shows that this estimate increased when the estimate of the association between the exposure and the outcome decreased, i.e., when reliability decreased, and sibling correlations increased.

**Fig. 4:**
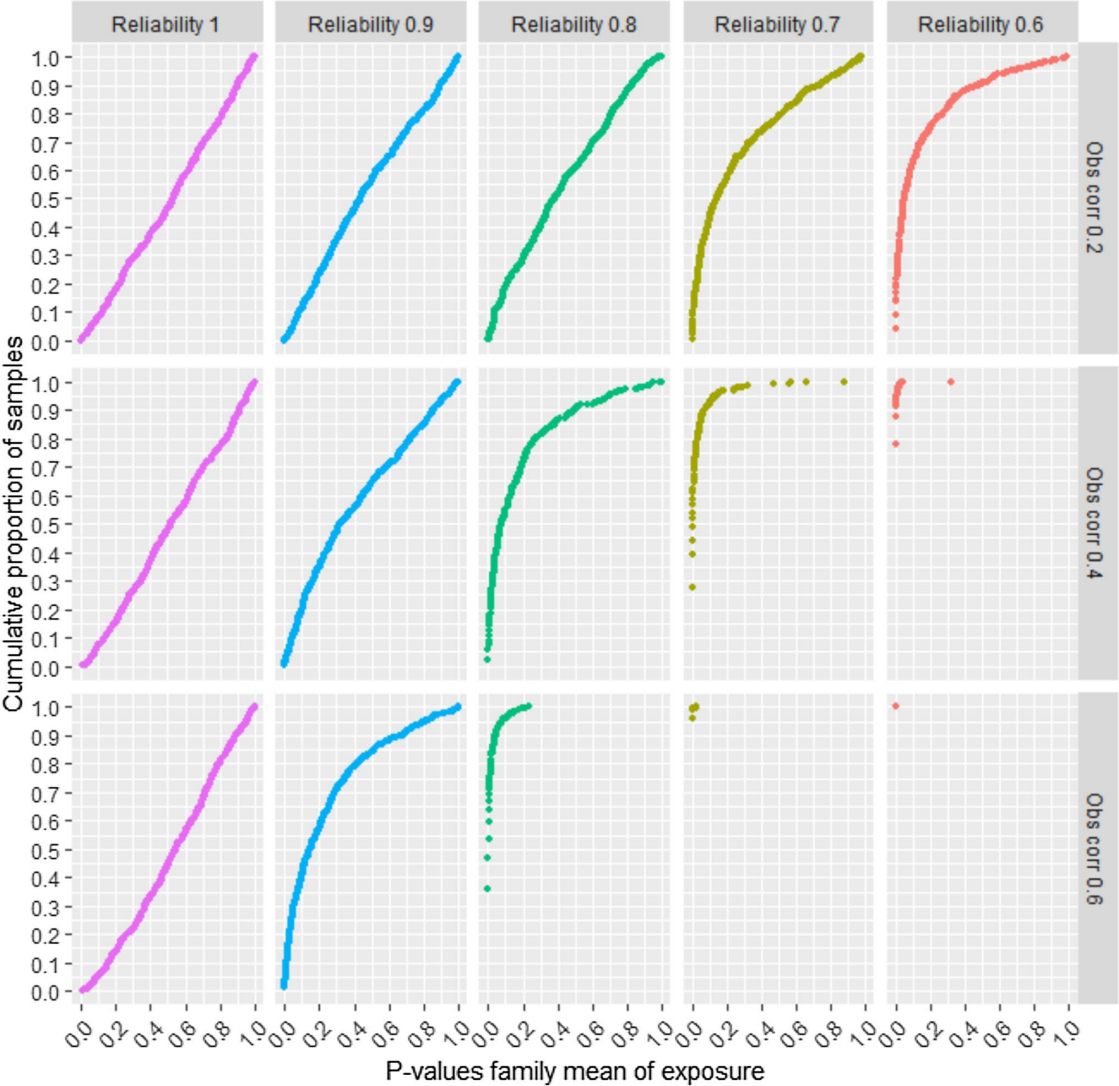
Risk of falsely concluding that familial confounding exists—ordinal single-item outcome—n = 2,000. Notes: The y-axis shows cumulative proportion of samples with different p-values for the association between the family mean of the exposure and the outcome. “Obs corr” is the observed correlation between two siblings’ exposure variables. “Reliability” is the reliability of the observed exposure variables. The outcome is a five-level symmetric single-item ordinal variable. Proportions in the five response categories of the outcome were: 3.6%, 23.8%, 45.2%, 23.8%, and 3.6%

We also examined the p-values of the association between the family mean of the exposure and the outcome. The y-axis in Fig. 4 shows the cumulative proportion of random samples with p-values up to any given value. The full range of p-values between 0.00 and 1.00 are shown. For example, Fig. 4 shows that about 90% of the samples provided a p-value of 0.05 or less for the association between the family exposure mean and the outcome, when reliability of the exposure was 0.7, the sibling correlation of the exposure was 0.4, and the sample size was n = 2,000. This implies that if a researcher decides that a p-value less than 0.05 should be interpreted as strong evidence for the existence of this association, there will be about 90% risk of drawing a false conclusion of familial confounding in this situation. This risk would be about 60% or 30% if *P* < 0.01 or *P* < 0.001 were used as evidence, respectively. When reliability was 0.6, and the sibling correlation of the exposure was 0.4, about 80% of the samples had p-values less than 0.001. Hence, there was about 80% risk of falsely concluding that familial confounding existed when using a p-value threshold of 0.001 in this situation. Figure 4 shows that the p-values for the association between the family exposure mean and the outcome decreased as reliability of the exposure went down, and sibling correlations went up. P-values are dependent on sample size, and results from different samples sizes (n = 500 and n = 5,000) are shown in Supplemental figures S1 to S4. The SibSim app allows researchers to examine a lot of different situations (including different sample sizes), and exact p-values and cumulative proportions of p-values are shown when hovering over the plot in the app with the mouse.

## Aggregate of several ordinal items as outcome

### Methods

The steps described above for generating the single-item outcome were then performed for the aggregate outcome variables. The difference was that five observed ordinal variables affected by the latent outcome were generated instead of one, and that the latent factor explained only 49% (0.7^2) of the variance of each of the five variables. The latter change was made to reflect the common situation where the items only partially reflect an underlying construct, which then cannot be measured with a single item. In the same way as with the single item outcome measure, each of these five outcome variables were categorized into five response categories, as shown in Table 1. Mean scores of these five ordinals were then calculated and used as outcome in linear models as if these aggregates were truly continuous. Symmetric ordinal variables were used in analyses presented in the paper, while the SibSim app also allows analysing moderately and extremely asymmetric outcome variables. The initial analysis model was run as a linear mixed effects model with the lme4 package [36] with a random intercept for family. The family mean of the exposure was added in the sibling control model.

### Results

**Fig. 5:**
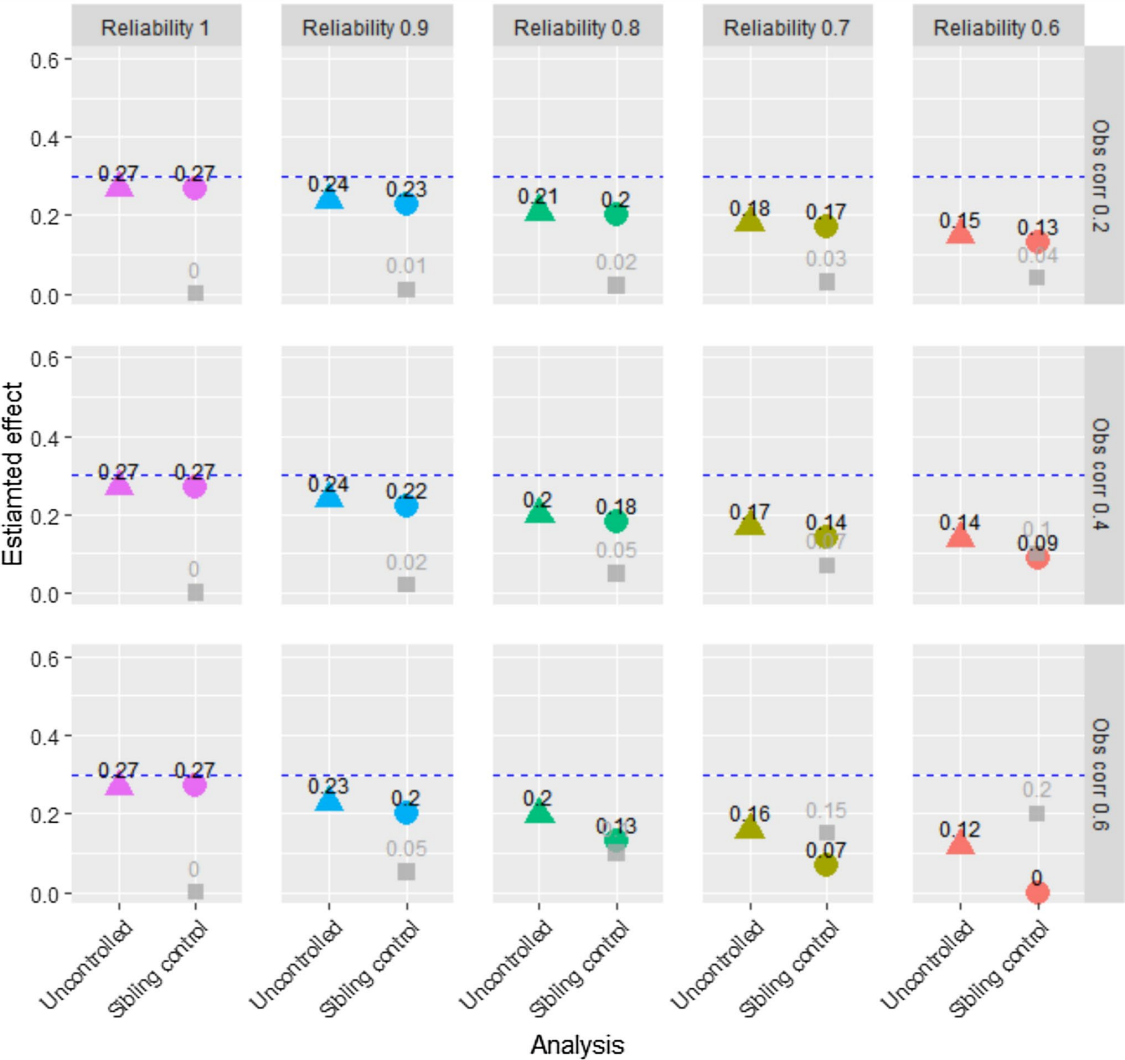
Results from uncontrolled and sibling control models—outcomes are aggregates of symmetric ordinals—n = 2,000. Notes: “Obs corr” is the observed correlation between two siblings’ exposure variables. “Reliability” is the reliability of the exposure variables. The outcome is the mean of five symmetric ordinal variables. Proportions in the five response categories of the outcome were: 3.6%, 23.8%, 45.2%, 23.8%, and 3.6%. The true causal effect was b=0.3, as indicated by the dotted line. Triangles represent the estimated association between the exposure and the outcome in an uncontrolled model. Circles represent the estimate of this association in the sibling control model. Gray squares represent the estimated association between the family mean of the exposure and the outcome. The true value of this latter association is zero

**Fig. 6:**
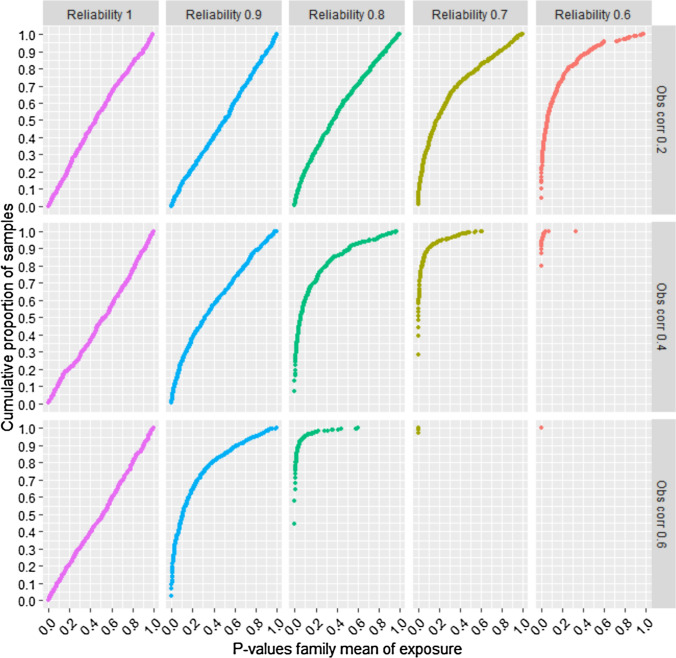
Risk of falsely concluding that familial confounding exists—outcomes are aggregates of symmetric ordinals—n = 2,000. Notes: The y-axis shows cumulative proportion of samples with different p-values for the association between the family mean of the exposure and the outcome. “Obs corr” is the observed correlation between two siblings’ exposure variables. “Reliability” is the reliability of the observed exposure variables. The outcome is the mean of five symmetric ordinal variables. Proportions in the five response categories of the outcome were: 3.6%, 23.8%, 45.2%, 23.8%, and 3.6%

Figure 5 shows results from using the mean of five symmetric ordinal variables as outcome. The exposure-outcome associations were somewhat attenuated even when reliability of the exposure was 1. This is due to the outcome being an aggregate of five indicators not perfectly correlated with the latent outcome factor. However, the estimates from the uncontrolled and the sibling control models were the same when there was no measurement error in the exposure. Also, the estimate of the association between the family mean of the exposure and the outcome was unbiased in this situation. As the reliability of the exposure went down and the sibling exposure-correlation went up, uncontrolled and sibling control models provided increasingly diverging results. Figure 5 also shows that the estimate of the association between the family mean of the exposure and the outcome increased when exposure reliability went down, and sibling correlations went up. Figure 6 shows that when reliability decreased and sibling correlations increased, the p-values of this association decreased. Figure 6 includes the full range of p-values between 0.00 and 1.00. For example, Fig. 6 shows that when reliability of the exposure was 0.7, the sibling correlation of the exposure was 0.4, and the sample size was n = 2,000, about 90% of the samples provided p-values below 0.05, about 80% of the samples provided p-values below 0.01, and about 45% of the samples provided p-values below 0.001 for the association between the family mean of the exposure and the outcome. Hence, the risk of falsely concluding that familial confounding existed would be about 90%, 80%, or 45% if p-values less than 0.05, 0.01, and 0.001, respectively, were interpreted as strong evidence for the association between the family mean of the exposure and the outcome. When reliability was 0.6, and the sibling correlation of the exposure was 0.4, about 90% of the samples had p-values less than 0.001. Hence, there would be about 90% risk of falsely concluding that familial confounding existed if a p-value threshold of 0.001 was used in this situation.

Results from analyses of different samples sizes (n = 500 and n = 5,000) are shown in Supplemental figures S5to S8.

## Additional analyses

**Fig. 7:**
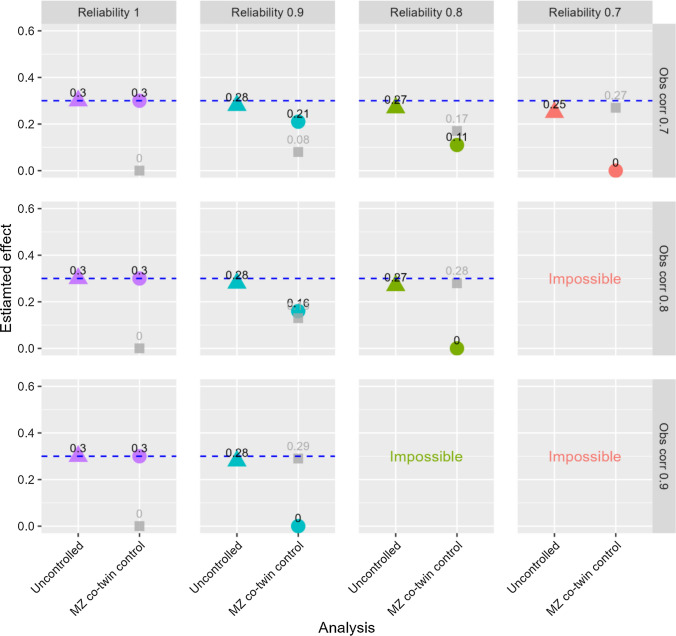
Results from uncontrolled and monozygotic co-twin control models—ordinal single-item outcome—n = 2,000. Notes: “Obs corr” is the observed correlation between two monozygotic twins’ exposure variables. “Reliability” is the reliability of the exposure variables. The outcome is a five-level symmetric single-item ordinal variable. Proportions in the five response categories of the outcome were: 3.6%, 23.8%, 45.2%, 23.8%, and 3.6%. The true causal effect was b=0.3, as indicated by the dotted line. Triangles represent the estimated association between the exposure and the outcome in an uncontrolled model. Circles represent the estimate of this association in the co-twin control model. Gray squares represent the estimated association between the family mean of the exposure and the outcome. The true value of this latter association is zero. “Impossible” refers to the fact that higher observed correlation than reliability implies a true correlation above one

**Fig. 8:**
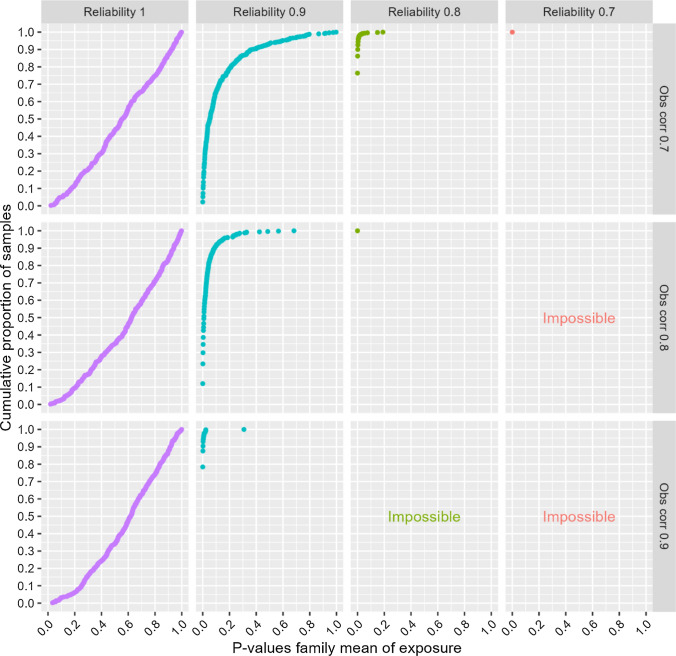
Risk of falsely concluding that familial confounding exists—ordinal single-item outcome—n = 2000. Notes: The y-axis shows cumulative proportion of samples with different p-values for the association between the family mean of the exposure and the outcome. “Obs corr” is the observed correlation between two monozygotic twins’ exposure variables. “Reliability” is the reliability of the observed exposure variables. The outcome is a five-level symmetric single-item ordinal variable. Proportions in the five response categories of the outcome were: 3.6%, 23.8%, 45.2%, 23.8%, and 3.6%. “Impossible” refers to the fact that higher observed correlation than reliability implies a true correlation above one

**Fig. 9:**
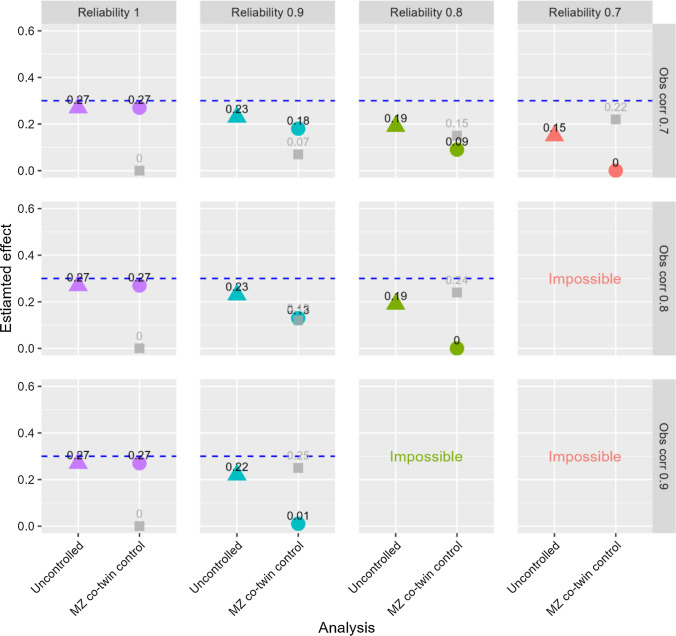
Results from uncontrolled and monozygotic co-twin control models—outcomes are aggregates of symmetric ordinals—n = 2,000. Notes: “Obs corr” is the observed correlation between two monozygotic twins’ exposure variables. “Reliability” is the reliability of the exposure variables. The outcome is the mean of five symmetric ordinal variables. Proportions in the five response categories of the outcome were: 3.6%, 23.8%, 45.2%, 23.8%, and 3.6%. The true causal effect was b=0.3, as indicated by the dotted line. Triangles represent the estimated association between the exposure and the outcome in an uncontrolled model. Circles represent the estimate of this association in the co-twin control model. Gray squares represent the estimated association between the family mean of the exposure and the outcome. The true value of this latter association is zero. “Impossible” refers to the fact that higher observed correlation than reliability implies a true correlation above one

**Fig. 10:**
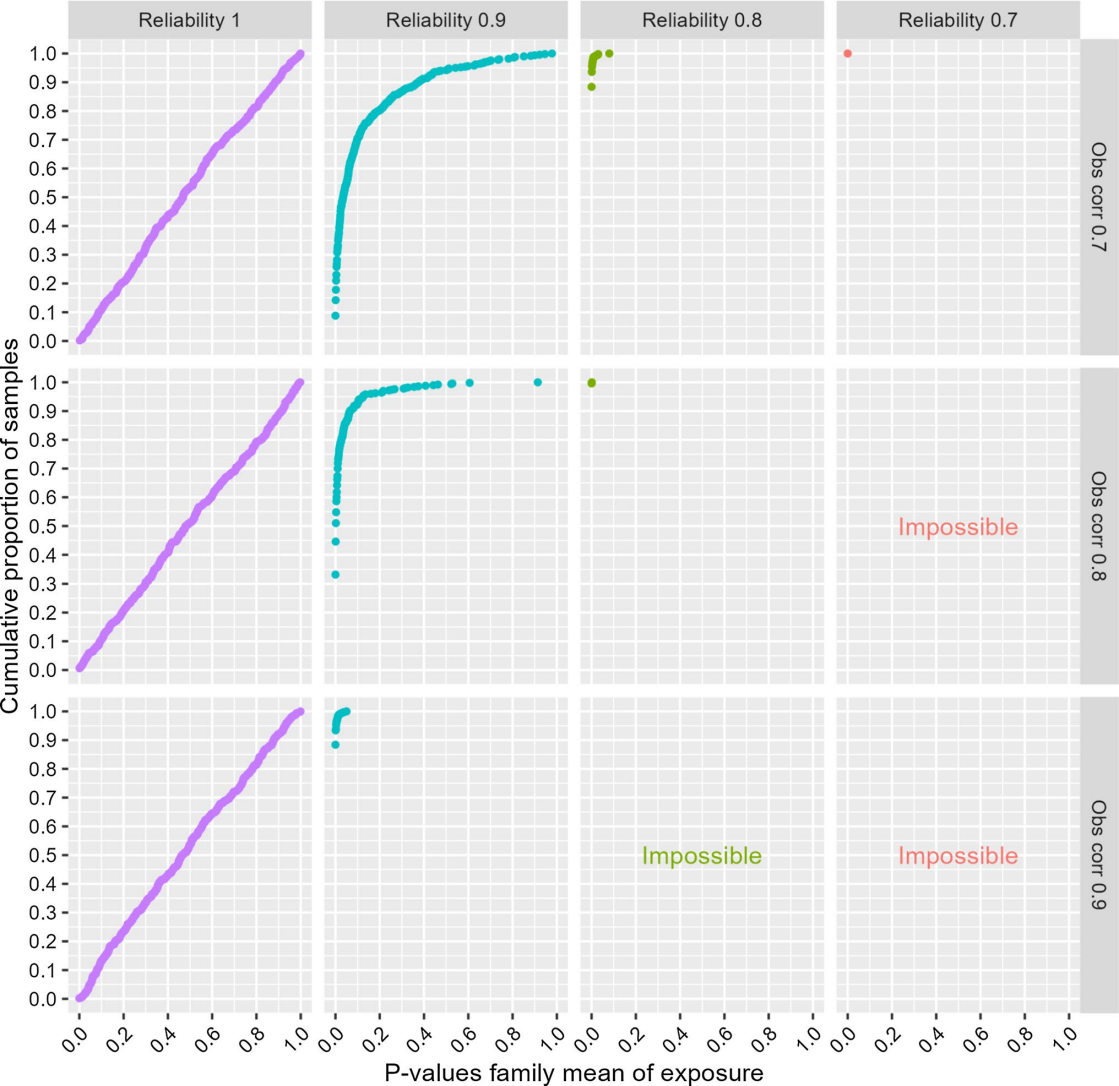
Risk of falsely concluding that familial confounding exists—outcomes are aggregates of symmetric ordinals—n = 2,000. Notes: The y-axis shows cumulative proportion of samples with different p-values for the association between the family mean of the exposure and the outcome. “Obs corr” is the observed correlation between two monozygotic twins’ exposure variables. “Reliability” is the reliability of the observed exposure variables. The outcome is the mean of five symmetric ordinal variables. Proportions in the five response categories of the outcome were: 3.6%, 23.8%, 45.2%, 23.8%, and 3.6%. “Impossible” refers to the fact that higher observed correlation than reliability implies a true correlation above one

*Higher exposure correlations in monozygotic twins:* Data with observed exposure correlations between 0.7 and 0.9 were generated to mimic studies using exposures highly correlated between monozygotic twins [30]. It is not meaningful to model reliabilities that are lower than the observed correlations, as that would imply true correlations above one. Reliabilities between 1 and 0.7 were therefore modelled in these analyses, and some combinations were not examined (i.e., reliability of 0.7 in combination with observed correlations of 0.8 or 0.9, and reliability of 0.8 in combination with observed correlations of 0.9). The results are shown in Figs. 7 and 8 for probit regression of a single ordinal outcome, and in Figs. 9 and 10 for linear regression of aggregates of several ordinals as outcome. The figures illustrate that exposure variables that are highly correlated between monozygotic twins may not be very useful in co-twin control analyses, unless the exposures are measured with almost perfect reliability. Even when reliability was 0.9, the estimates of the exposure-outcome association as well as the association between the family mean of the exposure and the outcome were clearly biased in all situations, and the risk of drawing false conclusions of familial confounding was high. For the probit analyses of the single-item outcome, there was about 80% or 25% risk of drawing a false conclusion of familial confounding (n = 2000) when using *P* < 0.05 or *P* < 0.001, respectively, as evidence for an association between the family exposure mean and the outcome when reliability was 0.9 and the observed exposure correlation 0.8. The corresponding numbers were about 90% and 50% for the linear analyses of the aggregate outcomes. An extended version of the SibSim app (SibSimExtended) allows examining such high exposure correlations relevant for some studies of monozygotic twins.

*Different effect sizes:* We wanted to explore if the current findings were only relevant for one specific effect size. Supplemental Figure S9 shows results when the true causal effect was b = 0.5. The outcome was a single-item symmetric ordinal variable, treated as categorical in a probit regression model. Supplemental Figure S10 shows the cumulative proportion of samples with different p-values for the estimated association between the family mean of the exposure and the outcome. Supplemental Figures S11 and S12 show results when the mean of five symmetric ordinal variables was treated as a continuous outcome in a linear model. The figures show the same pattern of results as when the true causal effect was b = 0.3: More divergent results from uncontrolled versus sibling control models, higher association estimates for the family mean of the exposure and the outcome, and lower p-values for this association, when the reliability of the exposure went down, and the sibling correlation of the exposure went up. The SibSim app allows examining a lot of different effect-sizes.

## The SibSim app

See Supplemental Information for details on the app.

## Discussion

The current study and the associated SibSim app provide results with practical importance for researchers conducting and reviewing sibling control studies. Such studies can be very useful for accounting for unmeasured familial confounding. However–like other designs–the sibling control design has its sources of bias, and measurement error in the exposure is one of them [5, 15]. The current results showed that associations between exposures and outcomes can be substantially attenuated in sibling control models, only due to measurement error in the exposure. The results also showed that as the estimated association between the exposure and the outcome was deflated, the association between the family mean of the exposure and the outcome was inflated, thus increasing the risk of falsely concluding that familial confounding existed.

The current study adds to previous knowledge by providing results quantifying the risk of falsely concluding that true causal effects are confounded in sibling control studies. The association between the family mean of the exposure and the outcome may be interpreted as evidence for familial confounding. The current results showed that p-values for this estimate were heavily affected by exposure reliability and by sibling-correlations in the exposure. Further, the results showed a substantial risk of falsely concluding that familial confounding existed even when using very conservative p-value thresholds for the association between the family mean of the exposure and the outcome. For example, in several situations there was more than 30% risk of falsely concluding that truly causal effects were confounded when interpreting p-values below 0.001 as strong evidence for this association. When using p-values below 0.05 or 0.01 as evidence, there was as much as about 90% and 60% risk, respectively, for drawing false conclusions of familial confounding in several situations. These findings are in accordance with previous warnings of the need to consider aspects of a study when interpreting p-values rather than relying on arbitrary cut-offs for categorizing p-values as significant or non-significant [20]. The current study adds to this by quantifying the risk of drawing false conclusions in sibling control studies when not adequately considering measurement error and sibling correlations in the exposure.

A main point from the current study is the importance of highly reliable exposure measures in sibling control studies. The results showed that even with reliability levels often considered good or acceptable (e.g., proportion of true to total variance of 0.8 or 0.7), estimates of exposure-outcome associations were substantially deflated in some situations, and estimates of the association between the family exposure mean and the outcome were correspondingly inflated. This emphasizes that not only p-values, but also reliability, should be assessed in the context of the study. A sibling control study may require more reliable exposure measures than other studies, and studies examining exposures that are relatively highly correlated between siblings (e.g., educational level) may require particularly reliable exposures. This may be particularly relevant for studies including monozygotic twins who share 100% of their genes, thus potentially implying more highly correlated exposure variables than among other siblings. Some studies have reported observed corelations in the range 0.7 to 0.9 between monozygotic twins’ variables [30]. Even if the researcher has reason to believe the exposures are measured with reliability as high as 0.90, the current results showed that there will be substantial risk of bias with exposure correlations of this magnitude.

In the situations where reliability was equally high as the observed exposure correlations, the true sibling or twin correlations were 1.0. Hence, there was no real exposure discordance, and the sibling or co-twin control analyses were pointless [15]. Such situations may also appear in real-life studies, but this will not be evident unless the researcher acknowledges the importance of assessing the reliability of the exposure and its implications for the true sibling discordance.

Researchers might consider increasing reliability of their measures by aggregating variables, such as using multi-item scales for constructing latent variables, measuring the exposure on several occasions, or using self-report in addition to reports from friends or partners, or by explicitly modelling measurement error in the exposure [37–40]. The current results on detrimental effects of measurement error and sibling correlations are in line with previous results on continuous outcomes in linear regression models and logistic regression models with binary outcomes [5]. Regarding linear models with identity links, the effect of measurement error and sibling correlations can be calculated and corrected [5, 15]. The current study adds to this by showing results for ordinal outcome variables commonly used in questionnaire studies.

The results from the analyses of aggregated ordinals as outcomes showed attenuated association estimates even at perfect reliability of the exposure variables. As discussed in the introduction, this illustrated situations where the items only partially reflect an underlying outcome construct. Estimates were equally attenuated in the uncontrolled and the sibling control model in this situation, and the estimate of the association between the family mean of the exposure and the outcome was unbiased. Thus, using a less than perfect measure of the outcome did not lead to results that typically would be falsely interpreted as evidence for familial confounding. Nevertheless, the findings emphasize the importance of reliable measures of the outcome to obtain unbiased association estimates in uncontrolled as well as sibling control models.

The current findings illustrate that interpreting results from sibling control studies may not be straightforward, i.e., what results should be interpreted as evidence for confounding and what results should be interpreted as evidence against confounding may not be self-evident. An association between the exposure and the outcome that is attenuated in a sibling control model in addition to the presence of an association between the family mean of the exposure and the outcome, may (falsely) be interpreted as evidence that the association between the exposure and the outcome is (partially) confounded by familial factors. Another approach may be to consider any remaining association between the exposure and the outcome in a sibling control model as evidence supporting a hypothesis of causality. The SibSim app associated with this paper provides a graph showing statistical power to detect true causal effects in sibling control studies after accounting for expected attenuation of the estimate due to measurement error in the exposure. Hence, researchers can consider the power to detect true causal effects of their exposure on their outcome and at the same time consider the risk of falsely concluding that family effects are present for any given combination of exposure reliability, sibling-correlation in the exposure, sample size, true effect size, type of outcome variable and level of symmetry/asymmetry in the outcome in a real-life study. It may be important to consider statistical power to detect remaining true effects when assessing the risk of falsely concluding that familial confounding exists. Large samples will increase the statistical power to detect attenuated true causal effects. However, large samples will also increase the risk of falsely identifying familial confounding that does not exist. Small samples will reduce statistical power to detect attenuated true causal effects, but at the same time reduce the risk of falsely concluding that family effects are present. Hence, researchers may come to different conclusions regarding familial confounding depending on the combination of sample size and whether they choose to depend their conclusions on observed associations between the family exposure mean and the outcome or on remaining observed associations between the exposure and the outcome in the sibling control model. The current results emphasize the importance of triangulation when assessing causality and confounding [41]. Different research designs approach unmeasured confounding in different ways [se examples in 42, 43, 44], and results from sibling control studies should be compared to results from other designs with other sources of bias.

Additional sources of bias in sibling control studies: There are several sources of bias in sibling control studies in addition to measurement error. Mediators with shared effects on siblings may introduce bias as they will also be controlled when adjusting for shared confounders [45]. Siblings discordant for an exposure may be more different regarding confounders than unrelated individuals are, implying that nonshared confounders can introduce bias. This has been demonstrated by Frisell and colleagues [5], and in a recent simulation study by Esen and colleagues [46]. The latter study provides graphical illustrations of bias under different levels of exposure and confounder correlations, thus guiding interpretation of results from sibling control studies. The current study and the study from Esen and colleagues [46] together show that highly correlated exposures may increase bias from several sources (i.e., measurement error and nonshared confounders) in sibling control studies. The studies by Frisell and Esen [5, 46] emphasize the need to make explicit a priori assumptions of associations between exposures, outcomes, and potential mediators and confounders when using sibling control studies, for example by constructing DAGs (directed acyclic graphs) [47]. Selection effects (i.e., sibling control studies consist of discordant siblings rather than the entire population) and carry over effects (e.g., one siblings’ exposure affects the other sibling’s outcome or vice versa) may also introduce bias [13, 48], thus adding to the need for making a priori assumptions explicit.

The current results quantify bias in studies using sibling pairs. The analysis methods can also be applied for higher numbers of siblings per family, by calculating the family mean from all the participating siblings’ exposure variables and controlling each sibling’s exposure for this mean [49]. An extended version of the SibSim app (SibSimExtended) allows defining a percentage of families participating with more than two siblings, to examine bias in these situations.

*Limitations*: The current study has several limitations that may reduce generalizability of the findings. First, even if we have examined several different scenarios, there may be other relevant situations not included here. To remedy this, we have developed the SibSim app where researchers can examine many additional situations. We believe this will ensure that researchers can obtain simulated results relevant for a wide variety of real-life situations. Nevertheless, even the app cannot cover all possible variations in real-life studies, and there will still be scenarios for which we do not provide relevant information. Second, real-life researchers may not know the reliability of their exposure measures, thus limiting the usefulness of the current findings. However, even if the researcher does not know the exact reliability of their measures, the current results can be used to examine the risk of falsely concluding that true effects are confounded given different potential levels of reliability in the exposures, thus allowing the researcher to discuss this explicitly when interpreting the results. Third, it should be noted that monozygotic co-twin control studies can be biased if the assumption of 100% shared genetics is violated due to de novo mutations affecting health outcomes [50, 51]. Genetic effects will then not be fully controlled. Fourth, the current study has only examined one of the designs taking advantage of the genetic information in samples including siblings and/or twins. Such samples are also used to establish the origin of de novo mutations [50], improve other designs relevant for causal inference, such as Mendelian randomization [52], and in the study of epigenetic effects on health [53], to name only a few. Monozygotic twins have also been used to establish the importance of prenatal environment for health outcomes, as even monozygotic twins do not fully share environmental exposures in utero, as indexed by discordance in birth weight [54].

*Conclusion*: Sibling control studies can be valuable for accounting for unmeasured familial confounding between risk factors and health outcomes within the field of epidemiology. However, sibling control studies may also introduce bias when there is measurement error in the exposure. The current study showed that results from such studies can be substantially biased even at relatively high levels of reliability in the exposure. Also, the results showed that as the exposure-outcome association was deflated due to measurement error, the association between the family mean of the exposure and the outcome got correspondingly inflated. This association may be interpreted as reflecting familial confounding. The p-value of this association may inform the researcher’s interpretation of its relevance, and the current study showed substantial risk of falsely concluding that true causal effects were confounded in several situations even with relatively conservative interpretations of p-values.

We have developed the SibSim app where epidemiological researchers can examine many different situations not included in the paper. The current paper and the associated app provide results with practical relevance for researchers conducting or reviewing sibling control studies and may contribute to reducing the risk of drawing false conclusions of familial confounding from such studies. This may increase the validity and utility of sibling control studies within the field of epidemiology in the future.

### Supplementary Information

Below is the link to the electronic supplementary material.

Supplementary file1 (DOCX 455 kb)
