# Supplementary material for: Familial confounding or measurement error? How to interpret findings from sibling and co-twin control studies
Source: Eur J Epidemiol. 2024 Jun 16;39(6):587–603. doi: 10.1007/s10654-024-01132-6 (PMC11249619; doi:10.1007/s10654-024-01132-6)
Supplement: Supplementary file 1 — Supplementary file1 (DOCX 455 kb) [file 10654_2024_1132_MOESM1_ESM.docx]

Supplementary information to

*Familial confounding or measurement error? How to interpret findings from sibling and co-twin control studies.*

1. **Supplemental methods**

**The SibSim app**

*What can it do?* [The SibSim app](http://kristin-gustavson.shinyapps.io/sibsim) performs the same simulations as those presented in the paper and can be used to examine a lot of additional situations. By hovering over the graphs in the app, the user will get exact numbers for p-values and cumulative proportions of samples.

The user can examine any combination of the following aspects: any sample size between 100 and 5,000, any size of the causal effect between b=0.1 and b=0.5, any reliability of the exposure between 0.6 and 1.0, and any observed sibling correlation in the exposure between 0.2 and 0.6. These choices can be made for single-item ordinal outcomes and aggregates of several ordinal outcomes, and the user can choose between symmetric, moderately asymmetric, and highly asymmetric outcome variables (see Table 1 in the manuscript for definitions of the different levels of asymmetry). An extended version of the app [(SibSimExtended)](http://kristin-gustavson.shinyapps.io/sibsimextended) allows examining exposure correlations between 0.2 and 1.0. Please note that some combinations of observed exposure correlations and exposure reliabilities will be impossible as they will imply true exposure correlations above one (e.g., observed correlation of 0.8 and exposure reliability of 0.7). The extended version also allows defining a percentage of the families participating with more than two siblings. Three siblings will then be modelled for these families.

Truly continuous outcomes may not be very common in questionnaire studies. Nevertheless, we have included this as an option in the SibSim app (both versions). Both versions of the app also provide cumulative proportions of samples with different p-values for the association between the exposure and the outcome in sibling-control models. This translates to statistical power to detect a true (but possibly attenuated) causal effect.

*More details on the app:* The app (both versions) was developed in RStudio [1] with the package shiny [2]. The app also uses the following packages: MASS [3], dplyr [4], lme4 [5], lmerTest [6], lmtest [7], shinybusy [8], ggplot2 [9], cowplot [10], and plotly [11].

*Where to find the app and its code:* The app is released online and does not require R or RStudio to use. Please use [this link for the basic version](http://kristin-gustavson.shinyapps.io/sibsim) of the app, [and this link for the extended version](http://kristin-gustavson.shinyapps.io/sibsimextended). The R code for both versions of the app is [available online at OSF.](https://osf.io/ryszk/?view_only=603f09956e904417be0ca0cce618a1d3)

**Standardizing regression coefficients from the ordered probit regression model:**

Regression coefficients from the ordered probit regression model can be interpreted as associations between the predictor and the underlying latent continuous outcome variable. Because the residual variance is fixed to one in probit models, adding new predictor variables to a model can potentially re-scale the regression coefficients as the total variance of the underlying outcome variable increases when explained variance increases. Therefore, regression coefficients from the ordered probit models were standardized with respect to the underlying continuous variable in the same way as in Gustavson and colleagues [12] and in accordance with Muthén and Muthén [13]. This was done in the following way:

b_std = (b*SD(x))/SDu

SDu = sqrt (variance explained by the predictors +1)

where SDu is the estimated standard deviation of the underlying latent variable, b_std is the standardized regression coefficient, b is the unstandardized regression coefficient, and SD(x) is the standard deviation of the predictor. When there is more than one predictor, the correlation between the two predictors needs to be taken into account when calculating explained variance.

1. **Supplemental results**

Probit models with single item-outcomes

*Different sample sizes:*

**Fig. S1** Results from uncontrolled and sibling control models – symmetric single-item outcome – n=500.


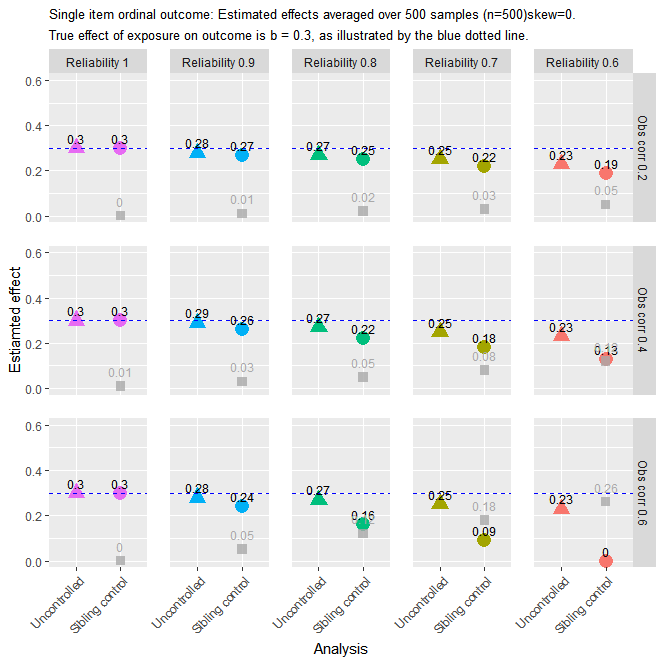


_Notes: “Obs corr” is the observed correlation between two siblings’ exposure variables. “Reliability” is the reliability of the exposure variables. The outcome is a five-level symmetric single-item ordinal variable._ _Proportions in the five response categories of the outcome were: 3.6%, 23.8%, 45.2%, 23.8%, and 3.6%. The true causal effect was b=0.3, as indicated by the dotted line. Triangles represent the estimated association between the exposure and the outcome in an uncontrolled model. Circles represent the estimate of this association in the sibling control model. Gray squares represent the estimated association between the family mean of the exposure and the outcome. The true value of this latter association is zero._

**Fig. S2** Risk of falsely concluding that familial confounding exists – symmetric single-item outcome – n=500


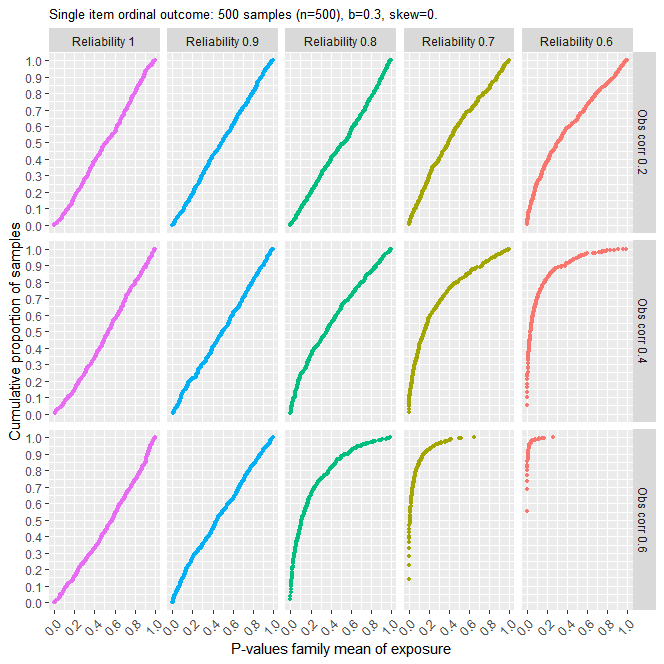


_Notes: The y-axis shows cumulative proportion of samples with different p-values for the association between the family mean of the exposure and the outcome. “Obs corr” is the observed correlation between two siblings’ exposure variables. “Reliability” is the reliability of the observed exposure variables. The outcome is a five-level symmetric single-item ordinal variable. Proportions in the five response categories of the outcome were: 3.6%, 23.8%, 45.2%, 23.8%, and 3.6%._

**Fig. S3** Results from uncontrolled and sibling control models – symmetric single-item outcome – n=5,000.
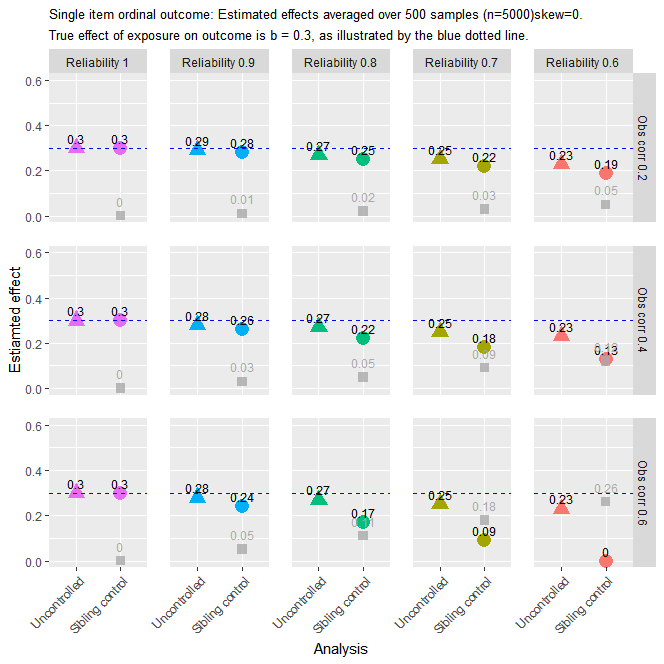


_Notes: “Obs corr” is the observed correlation between two siblings’ exposure variables. “Reliability” is the reliability of the exposure variables. The outcome is a five-level symmetric single-item ordinal variable. Proportions in the five response categories of the outcome were: 3.6%, 23.8%, 45.2%, 23.8%, and 3.6%._  _The true causal effect was b=0.3, as indicated by the dotted line. Triangles represent the estimated association between the exposure and the outcome in an uncontrolled model. Circles represent the estimate of this association in the sibling control model. Gray squares represent the estimated association between the family mean of the exposure and the outcome. The true value of this latter association is zero._

**Fig. S4** Risk of falsely concluding that familial confounding exists – symmetric single-item outcome – n=5,000
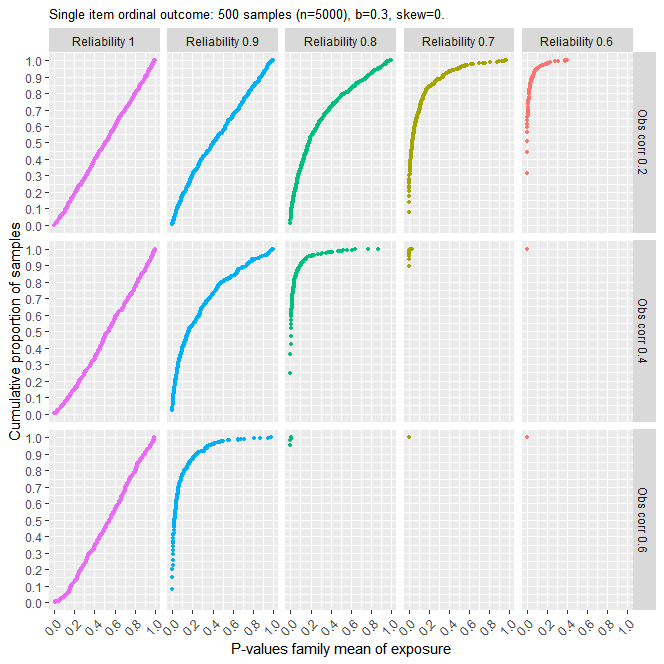


_Notes: The y-axis shows cumulative proportion of samples with different p-values for the association between the family mean of the exposure and the outcome. “Obs corr” is the observed correlation between two siblings’ exposure variables. “Reliability” is the reliability of the observed exposure variables. The outcome is a five-level symmetric single-item ordinal variable. Proportions in the five response categories of the outcome were: 3.6%, 23.8%, 45.2%, 23.8%, and 3.6%._

Linear models with aggregated outcomes

*Different sample sizes:*

**Fig. S5** Results from uncontrolled and sibling control models – outcomes are aggregates of symmetric ordinals – n=500


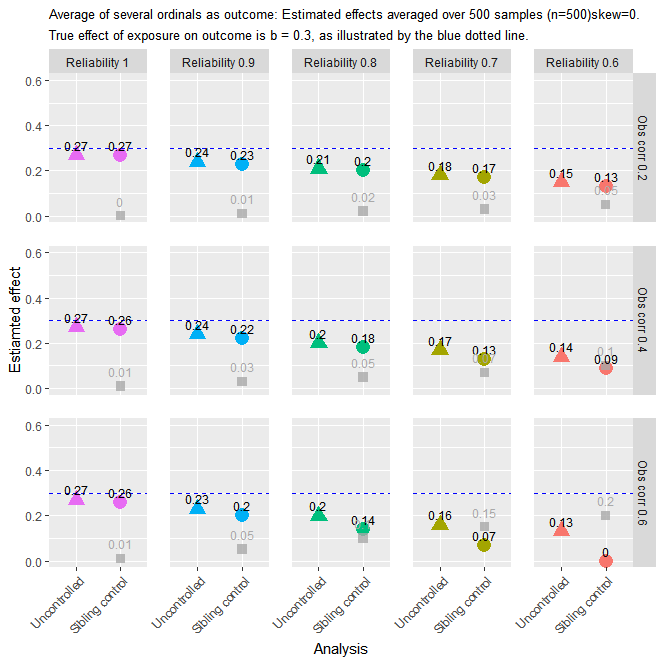


_Notes: “Obs corr” is the observed correlation between two siblings’ exposure variables. “Reliability” is the reliability of the exposure variables. The outcome is the mean of five symmetric ordinal variables._ _Proportions in the five response categories of the outcome were: 3.6%, 23.8%, 45.2%, 23.8%, and 3.6%. The true causal effect was b=0.3, as indicated by the dotted line. Triangles represent the estimated association between the exposure and the outcome in an uncontrolled model. Circles represent the estimate of this association in the sibling control model. Gray squares represent the estimated association between the family mean of the exposure and the outcome. The true value of this latter association is zero._

**Fig. S6** Risk of falsely concluding that familial confounding exists – outcomes are aggregates of symmetric ordinals -n=500.


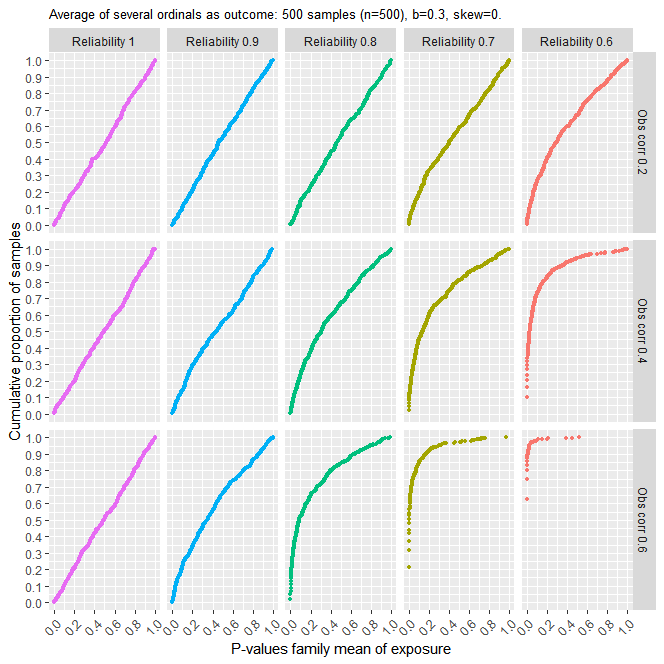


_Notes: The y-axis shows cumulative proportion of samples with different p-values for the association between the family mean of the exposure and the outcome. “Obs corr” is the observed correlation between two siblings’ exposure variables. “Reliability” is the reliability of the observed exposure variables. The outcome is the mean of five symmetric ordinal variables._ _Proportions in the five response categories of the outcome were: 3.6%, 23.8%, 45.2%, 23.8%, and 3.6%._

**Fig. S7** Results from uncontrolled models and sibling control models – outcomes are aggregates of symmetric ordinals – n=5,000


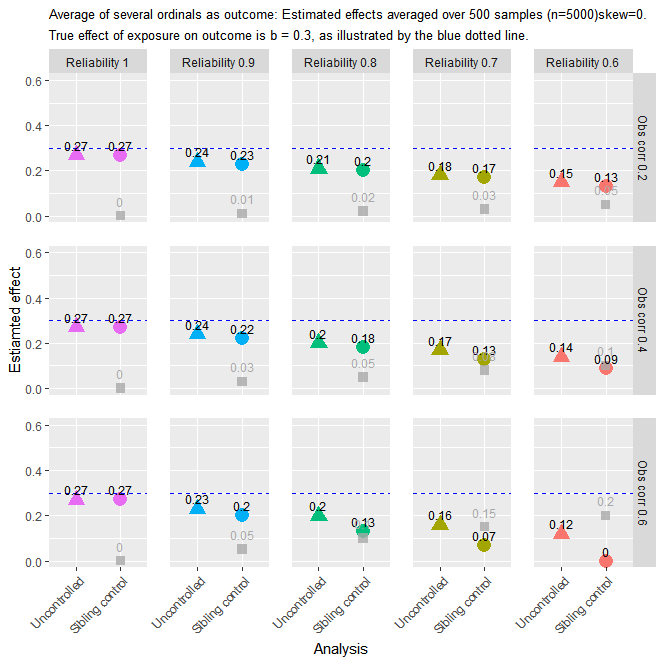


_Notes: “Obs corr” is the observed correlation between two siblings’ exposure variables. “Reliability” is the reliability of the exposure variables. The outcome is the mean of five symmetric ordinal variables._ _Proportions in the five response categories of the outcome were: 3.6%, 23.8%, 45.2%, 23.8%, and 3.6%. The true causal effect was b=0.3, as indicated by the dotted line. Triangles represent the estimated association between the exposure and the outcome in an uncontrolled model. Circles represent the estimate of this association in the sibling control model. Gray squares represent the estimated association between the family mean of the exposure and the outcome. The true value of this latter association is zero._

**Fig. S8** Risk of falsely concluding that familial confounding exists – outcomes are aggregates of symmetric ordinals – n=5,000.


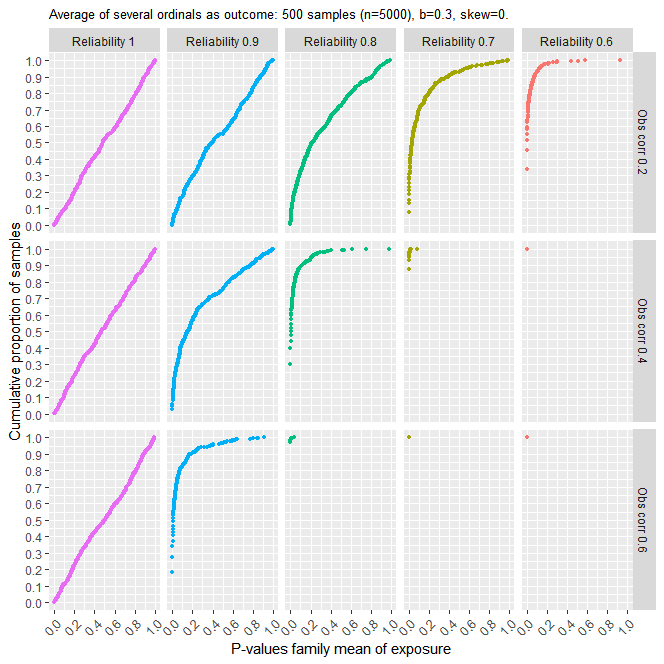
 _Notes: The y-axis shows cumulative proportion of samples with different p-values for the association between the family mean of the exposure and the outcome. “Obs corr” is the observed correlation between two siblings’ exposure variables. “Reliability” is the reliability of the observed exposure variables. The outcome is the mean of five symmetric ordinal variables._ _Proportions in the five response categories of the outcome were: 3.6%, 23.8%, 45.2%, 23.8%, and 3.6%._

Additional analyses

*Different effect sizes:*

**Fig. S9** Results from uncontrolled and sibling control models – symmetric single-item outcome – n=2,000.


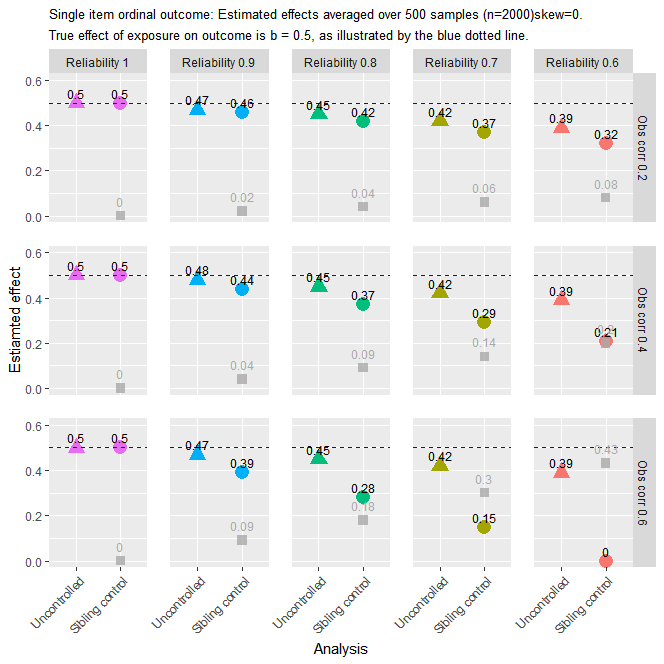


_Notes: “Obs corr” is the observed correlation between two siblings’ exposure variables. “Reliability” is the reliability of the exposure variables. The outcome is a five-level symmetric single-item ordinal variable._ _Proportions in the five response categories of the outcome were: 3.6%, 23.8%, 45.2%, 23.8%, and 3.6%. The true causal effect was b=0.5, as indicated by the dotted line. Triangles represent the estimated association between the exposure and the outcome in an uncontrolled model. Circles represent the estimate of this association in the sibling control model. Gray squares represent the estimated association between the family mean of the exposure and the outcome. The true value of this latter association is zero._

**Fig. S10** Risk of falsely concluding that familial confounding exists – symmetric single-item outcome – n=2,000.


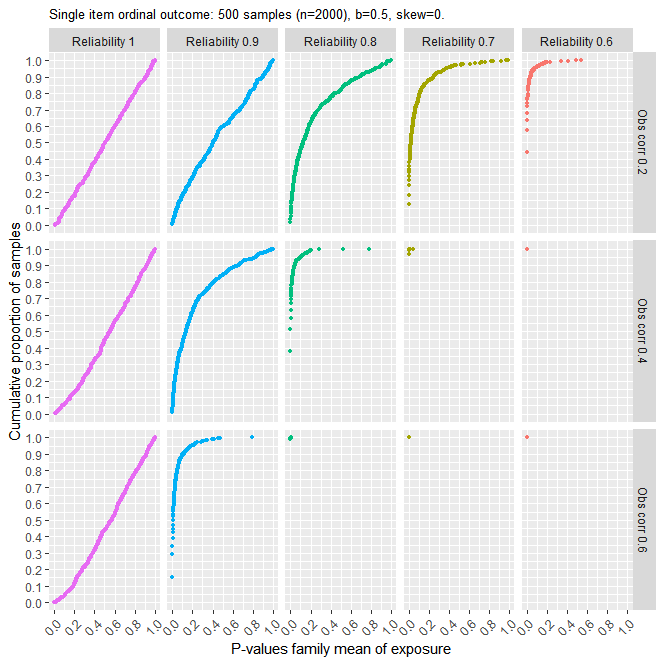


_Notes: The y-axis shows cumulative proportion of samples with different p-values for the association between the family mean of the exposure and the outcome. “Obs corr” is the observed correlation between two siblings’ exposure variables. “Reliability” is the reliability of the observed exposure variables. The outcome is a five-level symmetric single-item ordinal variable. Proportions in the five response categories of the outcome were: 3.6%, 23.8%, 45.2%, 23.8%, and 3.6%. The true effect of the exposure on the outcome was b=0.5._

**Fig. S11** Results from uncontrolled and sibling control models – outcomes are aggregates of symmetric ordinals – n=2,000.


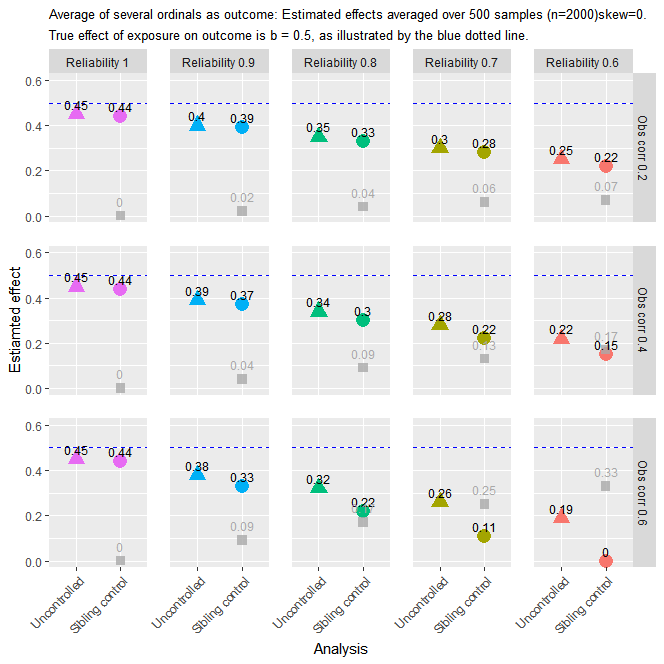


_Notes: “Obs corr” is the observed correlation between two siblings’ exposure variables. “Reliability” is the reliability of the exposure variables. The outcome is the mean of five symmetric ordinal variables._ _Proportions in the five response categories of the outcome were: 3.6%, 23.8%, 45.2%, 23.8%, and 3.6%. The true causal effect was b=0.5, as indicated by the dotted line. Triangles represent the estimated association between the exposure and the outcome in an uncontrolled model. Circles represent the estimate of this association in the sibling control model. Gray squares represent the estimated association between the family mean of the exposure and the outcome. The true value of this latter association is zero._

**Fig. S12** Risk of falsely concluding that familial confounding exists – outcomes are aggregates of symmetric ordinals – n=2,000.


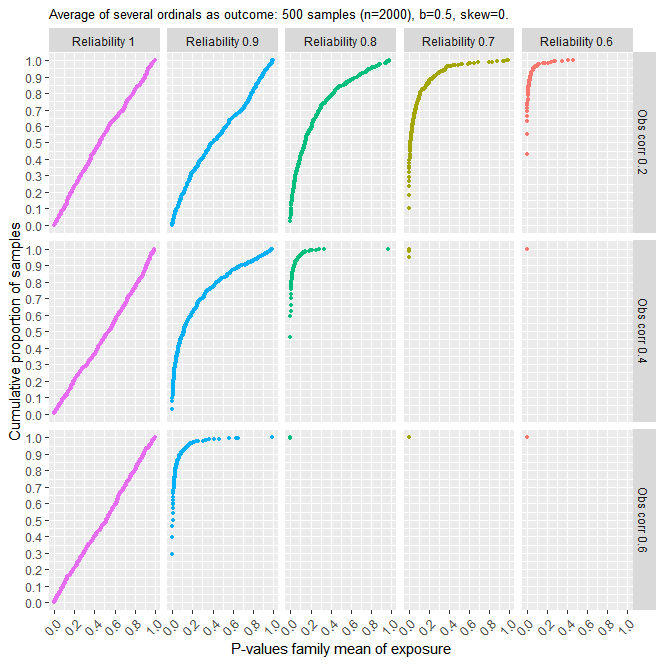


_Notes: The y-axis shows cumulative proportion of samples with different p-values for the association between the family mean of the exposure and the outcome. “Obs corr” is the observed correlation between two siblings’ exposure variables. “Reliability” is the reliability of the observed exposure variables. The outcome is the mean of five symmetric ordinal variables._ _Proportions in the five response categories of the outcome were: 3.6%, 23.8%, 45.2%, 23.8%, and 3.6%. The true effect of the exposure on the outcome was b=0.5._

**References**

1. RStudio Team, *RStudio: Integrated Development for R.* 2020, Boston, MA: RStudio, PBC.

2. Chang, W., et al., *shiny: Web Application Framework for R. R package version 1.8.0.9000*. 2023.

3. Venables, W.N. and B.D. Ripley, *Modern Applied Statistics with S*. Fourth ed. 2002, New York: Springer.

4. Wickham, et al., *dplyr: A Grammar of Data Manipulation. R package version 1.1.4*. 2023.

5. Bates, D., et al., *Fitting Linear Mixed-Effects Models Using lme4.* Journal of Statistical Software, 2015. **67**(1): p. 1-48.

6. Kuznetsova, A., P.B. Brockhoff, and R.H.B. Christensen, *lmerTest Package: Tests in Linear Mixed Effects Models.* Journal of Statistical Software, 2017. **82**(13): p. 1-26.

7. Zeileis, A. and T. Hothorn, *Diagnostic Checking in Regression Relationships.* R News, 2002. **2**(3): p. 7-10.

8. Meyer, F. and V. Perrier, *shinybusy: Busy Indicators and Notifications for 'Shiny' Applications. R package version 0.3.2*. 2023.

9. Wickham, H., *ggplot2: Elegant Graphics for Data Analysis Elegant Graphics for Data Analysis*. 2016, New York: Springer-Verlag

10. Wilke, C.O., *Streamlined Plot Theme and Plot Annotations for 'ggplot2'*. 2017.

11. Sievert, C., *Interactive Web-Based Data Visualization with R, plotly, and shiny*. 2020, Chapman and Hall/CRC.

12. Gustavson, K., E. Roysamb, and I. Borren, *Preventing bias from selective non-response in population-based survey studies: findings from a Monte Carlo simulation study.* Bmc Medical Research Methodology, 2019. **19**.

13. Muthén, L.K. and B.O. Muthén *Regression Analysis, Exploratory Factor Analysis, Confirmatory Factor Analysis, And Structural Equation Modeling For Categorical, Censored, And Count Outcomes*. http://www.statmodel.com, 2009.
